# Association of School Environment and After-School Physical Activity with Health-Related Physical Fitness among Junior High School Students in Taiwan

**DOI:** 10.3390/ijerph14010083

**Published:** 2017-01-15

**Authors:** Kai-Yang Lo, Min-Chen Wu, Shu-Chin Tung, City C. Hsieh, Hsueh-Hua Yao, Chien-Chang Ho

**Affiliations:** 1 Center for General Education, National Sun Yat-sen University, Kaohsiung City 804, Taiwan; luosun@ms25.hinet.net; 2 Office of Physical Education, Chung Yuan Christian University, Taoyuan City 320, Taiwan; minchenwu.cycu@gmail.com; 3 Department of Health and Leisure Management, Yuanpei University of Medical Technology, Hsinchu City 300, Taiwan; sctun@mail.ypu.edu.tw; 4 Department of Physical Education, National Tsing Hua University, Hsinchu City 300, Taiwan; chsieh@mail.nhcue.edu.tw; 5 Department of Radiological Technology, Yuanpei University of Medical Technology, Hsinchu City 300, Taiwan; yaohsuehhua@gmail.com; 6 Department of Physical Education, Fu Jen Catholic University, New Taipei City 242, Taiwan

**Keywords:** school environment, after-school physical activity, health-related physical fitness, adolescent

## Abstract

The relationship between students’ school environment and exercise habits is complex, and is affected by numerous factors. However, the few studies that have been conducted on this relationship have reported inconsistent results, especially regarding Taiwanese students. We conducted this cross-sectional study to investigate the association of school environment and after-school physical activity with health-related physical fitness in Taiwanese adolescents. Data were drawn from a national survey conducted by the Ministry of Education in Taiwan in 2008 of health-related physical fitness measurements among junior high school students (649,442 total) in grades seven to nine. School environment (level of urbanization, school size, presence of sports field or gymnasium) and after-school physical activity were assessed for their association with adolescents’ physical fitness measurements (body mass index (BMI), bent-leg sit-ups, 800-/1600-m run, sit-and-reach, standing long jump). Urban boys and girls perform significantly better in muscle strength and endurance, cardiorespiratory endurance, flexibility, and explosive power; girls from rural areas exhibited significantly worse scores in body composition. Boys from large-size schools performed the worst in cardiorespiratory endurance, flexibility, and explosive power; whereas girls from large-size schools performed the worst in muscle strength, muscle endurance, and explosive power, but had the best score for body composition. However, the differences in body composition of boys from large-, medium-, and small- size schools did not reach a statistically significant level. Adolescents of both genders in schools with a sports field or gymnasium exhibited significantly better in muscle strength and endurance, cardiorespiratory endurance, and explosive power. Boys in schools with a sports field or gymnasium had significantly better body composition; girls in schools with sports field or gymnasium differed significantly in flexibility. Adolescents of both genders who participated in physical activity after school had significantly better body composition, cardiorespiratory endurance, and flexibility. Boys who participated in physical activity after school significantly differed in explosive power, whereas girls who participated in physical activity after school exhibited significantly better flexibility. Thus, the current study demonstrated that some factors, including urbanization (school location in rural or urban areas), school size, school facility provision (school with or without sports fields or gymnasiums), and after-school physical activity participation are more important than others in shaping adolescents’ physical fitness in Taiwan; meanwhile, these association patterns differed by gender.

## 1. Introduction

Lack of regular physical activity is a well-documented risk factor for cardiovascular disease, hypertension, high cholesterol, obesity, and other chronic diseases [1,2,3]. Studies conducted in Taiwan have suggested that adverse health conditions may originate in childhood and adolescence [4,5]. The physical activity of adolescents is affected by a variety of factors, including age, gender, self-esteem, residential area, peer influences, parenting styles, their family’s socioeconomic status, and ethnic disparities [6].

Physical fitness is interpreted as a measure of the ability to perform physical activity that integrates the majority of bodily functions, and its components are divided into those related to health and those related to athletic skill [7]. Several studies have demonstrated that more active school-age youth have better cardiorespiratory endurance, muscle strength, muscular endurance, flexibility, and body composition than their less active counterparts [8,9,10]. These findings suggest a link between physical activity and health-related physical fitness. Therefore, physical fitness is an important issue for the health promoting school programs implemented by the Taiwan Ministry of Education. The Taiwan Ministry of Education conducted a series of nationwide surveys that evaluated the health-related physical fitness of students in elementary, junior, and high schools [11,12]. The results of the survey provide a preliminary reference regarding the health-related physical fitness of children and adolescents in Taiwan. Adolescents spend considerable quantities of time at school, and it is therefore essential to understand what opportunities they are given for school-based physical activity. To improve the physical fitness of school-age children and adolescents, the Taiwan Ministry of Education recommended that students exercise three times per week for 30 min, raising their heart rate to 130 bpm. Liu and Hsieh [13] emphasized a more practical approach involving accumulating intermittent physical activity for more than 10 min per time multiple times throughout the day. To monitor and improve adolescents’ physical function and health status, a national recommendation on the health-related physical fitness of Taiwanese students must be established that can be applied in counseling and education.

Chen et al. [14] investigated the prevalence of overweight and obesity in nationally representative samples of 6–18-year-olds, using the National Physical Fitness Survey (NPFS) conducted by the National Council on Physical Fitness and Sports in Taiwan in 1999, repeated in 2001. In 2007, the NPFS offered another opportunity to study the interrelationships between weight, hypertension, and health-related physical fitness measurements as early indicators of ill health.

Evidence indicates that sufficient school facilities for physical activity—such as sports fields and gymnasiums—promote physical activity in adolescents [15]. Thus, the lower levels of physical activity of adolescents enrolled in large-size schools might be related to poor facility provision. However, no previous study in Taiwan has investigated how students’ physical activity or fitness is related to school size. In addition, the after-school period is considered a “critical interval” that defines the propensity of adolescents for physical activity [16]. The after-school period may represent significant opportunities for educators and researchers to modify exercise habits and enhance physical activity for adolescents reported by Pelclová et al. [17]. In this study, we performed a cross-sectional investigation of environmental variables and health-related physical fitness, measuring five physical fitness indicators in Taiwanese adolescents aged 13–15 years. The aim of this study was to gather essential information for use in health policy reform concerning the health-related physical fitness of Taiwan’s adolescents.

## 2. Materials and Methods

### 2.1. Participants

The data consisted of 649,442 Taiwanese adolescents (*N* = 312,365 girls and *N* = 337,077 boys) in the seventh to ninth grades of junior high school, and were collected between February and June 2008. Students with a disability were excluded, because of the difficulty in the conducting health-related physical fitness measurements of these students. The measurements were made by school physical education teachers and nurses, all of whom had attended a regional training seminar and passed a certification test on standardized procedures. The protocols to be used were described in detail in a technical manual provided to all the junior high schools and participating teachers and nurses.

Both parents and students were informed about the aims and procedures of this study, and written informed consent was obtained from parents or guardians, as well as students providing their assent. The study was performed in accordance with the Helsinki Declaration of 1975 with regard to the conduct of clinical research, and was approved by the Ethical Committee of Ministry of Education in Taiwan (SAHIARC-MOE 104004).

### 2.2. Data Collection

The investigating variables—school environment (urbanization, school size, and school facility provision) and after-school physical activity—are described as follows.

#### 2.2.1. Urbanization

Urbanization was determined according to the urban–rural classification established by the Ministry of Education in Taiwan, in which population density, age composition, mobility, economic activity and family income, educational level, and health and sanitation facilities were considered. The urban group consists of adolescents whose schools are located in the three largest cities in Taiwan (i.e., Taipei, Taichung, and Kaohsiung Cities). All non-urban adolescents studying in the schools in remote rural areas are classified as the rural group.

#### 2.2.2. School Size

School size was determined based on the number of classes (i.e., the number of administrative groups of students) in each of the Taiwanese junior high schools for the grades seven to nine. A larger-size school was defined as one with 49 or more classes, a medium-size school as one with 13–48 classes, and a small-size school as one with fewer than 12 classes. The number of students for each class in junior high school ranged from 20 to 34, according to guidelines for class size in Taiwan.

#### 2.2.3. School Facility Provision

School facility provision was determined according to whether schools had a sports field and gymnasium, and was based on the 2008 annual report of physical education statistics.

#### 2.2.4. After-School Physical Activity Participation

In contrast with physical education classes at school, after-school physical activity was available to students with access to out-of-school sports facilities or home exercise equipment. After-school physical activity participation was determined based on whether the school’s students exercised after school. The frequency of after-school physical activity participation was defined as being more than one time per week. The recommended target for time spent in moderate to vigorous leisure-time physical activity was over 30 min after the normal school day. The Taiwan Ministry of Education recommended that the exercise frequency for students is three times per week with an intensity of heart rate of 130 bpm for 30 min per exercise session (i.e., moderate to vigorous exercise intensity). To fulfill the recommendation, each student in this study was required to participate in a physical education class twice per week during school days in addition to engaging in after-school physical activity more than once. The data used were determined using the 2008 annual report of physical education statistics.

### 2.3. Health-Related Physical Fitness Measurements

The following five health-related physical fitness measurements were taken according to the governmental guideline indicated on the official website of the Taiwan Ministry of Education [18]: body composition (body mass index, BMI), muscle strength and endurance (bent-leg sit-ups), explosive power (standing long jump), flexibility (sit-and-reach), and cardiorespiratory endurance (800-/1600-m run). The measurements were performed by school physical education teachers and nurses with the appropriate training. Inspectors from the Taiwan Ministry of Education and universities visited each site to supervise progress and adherence to standards.

#### 2.3.1. Body Composition

Body composition was measured using BMI. Participants took off their shoes and wore light clothes prior to the measurement of their height and weight. BMI was calculated as their weight in kilograms divided by the square of their height in meters (kg/m^2^). Weight and height were measured using a stadiometer and a digital or balance beam scale. All instruments were validated and approved by the Taiwan Bureau of Standards, Metrology, and Inspection.

#### 2.3.2. Muscle Strength and Endurance

Muscle strength and endurance was measured as the number of bent-leg sit-ups a student was able to perform within 1 min. Each participant lay supine on a mat, with knees bent at right angles and their hands crossed on the chest. A technician held the participant’s ankles firmly for support and conducted the count. The participant’s elbows had to touch the corresponding knee (i.e., left touch left, etc.). After each upward movement, the shoulders returned to touch the mat, but touching the mat with the head was not required.

#### 2.3.3. Explosive Power

Explosive power was measured through a standing long jump. The participants stood on a starting line and were instructed to jump forward with both feet as far as possible. They were free to use their arms and legs for countermovement. The distance from the starting line to the heel of the foot closest to the line was recorded. The longer distance from two attempts was recorded in centimeters.

#### 2.3.4. Flexibility

Flexibility was measured using the standard sit-and-reach test, scored by the most distant point reached with the fingertips on a ruler. The device used had a measuring scale positioned such that reaching the feet with the fingertips recorded a distance of 25 cm. Before the test, shoes were removed and participants were instructed to slowly reach forward as far as possible with their knees fully extended. The longer distance from two attempts was recorded in centimeters.

#### 2.3.5. Cardiorespiratory Endurance

Cardiorespiratory endurance was measured through an 800-m (for girls) or 1600-m (for boys) run. Adequate warm-up exercise was performed before the test. The participants ran in groups of six to eight from behind a starting line, and were instructed to try to keep a steady speed, finishing the run as quickly as possible.

### 2.4. Statistical Analysis

All data were processed using Statistical Package for the Social Science (SPSS) software (SPSS 22.0 version for Windows, SPSS Inc., Chicago, IL, USA). Descriptive statistics including means and standard deviations (SDs) were calculated for all variables. The effects of urbanization, school size, school facility provision, and after-school physical activity on health-related physical fitness measurements were evaluated using the Student’s *t* test or analysis of variance, with the significance level being set at 0.05. When a significant *p* value was found (*p* < 0.05), Tukey’s post hoc test was performed to determine the differences between the mean pairs. All values in tables are displayed as the means ± SD.

## 3. Results

### 3.1. Participant Demographics

Table 1 presents the participant demographics of the sample, including a breakdown of their age and gender. A total of 312,365 girls and 337,077 boys from the seventh through ninth grades in junior high schools participated in this study.

### 3.2. Association of Urbanization and School Size with Health-Related Physical Fitness

Of the participants, 7.08% of the boys (*N* = 21,101) and 6.87% of the girls (*N* = 19,249) lived in rural areas (Table 2). The BMI values of the boys in rural areas were comparable to those of the boys in urban areas. Girls who attended rural schools, however, had significantly higher BMI values than those who attended urban schools (20.05 ± 2.96 vs. 19.85 ± 2.91 kg/m^2^, *p* < 0.05). No significant difference was found in the BMI of boys attending schools of varying size (Table 2). Girls attending small-size schools, however, were discovered to have a significantly higher BMI (*p* < 0.05)—a trend comparable to that for schools in rural and urban areas.

Significant differences in physical fitness between small and large-size schools for both boys and girls were discovered. Boys from small-size schools were also found to have better performance at the 800-/1600-m run, sit-and-reach, and standing long jump than did those from large-size schools. Girls from small-size schools had better performance at the standing long jump, but could not reach as far in the sit-and-reach as girls from large-size schools did.

### 3.3. Association of School Facility Provision and After-School Physical Activity with Health-Related Physical Fitness

Of the junior high schools investigated in this study, approximately 70% and 90% had a sports field or indoor gymnasium, respectively (Table 3). Boys in schools with access to a sports field performed significantly better in the physical fitness tests than those without access to a sports field, except for the flexibility test. Girls in schools with access to a sports field had significantly higher muscular endurance, explosive power, and cardiorespiratory ability than did those in schools without, but significantly lower flexibility and similar BMI values. Similar results were obtained from boys and girls in the indoor gymnasium.

Approximately one-quarter (26%) of students took exercise after school (Table 3). The BMI and flexibility of adolescents of both genders who participated in exercise after school were better than those of adolescents who did not. The other three items relevant to physical fitness had inconsistent results between genders.

## 4. Discussion

From analysis of our data, we would first recommend the definition of national objectives for the health-related physical fitness of adolescents. Second, the conduction of a national surveillance study wherein the health-related physical fitness of adolescents is measured more precisely and consistently is suggested. Finally, the factors that may affect the health-related physical fitness of Taiwanese adolescents should be evaluated. This study recruited a total of 312,365 girls and 337,077 boys from the seventh through ninth grades of junior high schools in Taiwan. Of the students, 7.08% of boys and 6.87% of girls lived in rural areas. The BMI values of the boys living in rural areas were comparable to those of the boys living in urban areas. However, girls attending rural schools had significantly higher BMI values than those of girls attending urban schools (*p* < 0.05).

A great deal of effort was applied to comparing the physical fitness performance of rural and urban adolescents in Taiwan. We discovered that, for both genders, students in rural schools performed better in physical fitness tests than did those attending urban schools. These findings could be due to differences of age groups and gender differences. Students’ physical activity levels decreased with age, and boys had higher levels of physical fitness than did girls [4,19,20]. This gender difference could be due to the different stages of physical and psychological development of the boys and girls, and the different gender expectations they face [21,22]. Boys in Taiwan are encouraged to participate in sports-related activities more often than girls are [23], and are more competent at physical activities [19,20].

The current results are not consistent with those reported by Huang et al. [24], who indicated that no relationship exists between residential area and students’ physical activity levels. Another study identified that urban adolescents have higher physical activity levels than rural adolescents [25], possibly because urban adolescents have more access to sport facilities and more choices for recreational and leisure activities. Students’ age, sample sizes, different measures of physical fitness, and geographical locations may also be factors. Huang et al. [26] reported no significant difference in walking ability that may be improved physical fitness was found between adolescents from urban and rural areas. However, urban adolescents were found to have greater access to places suitable for physical fitness activities. Urban adolescents engaged in more physical activity after school, on holidays and weekends, and in total compared with rural adolescents.

Confirming the results of our study, Chen [27] reported that adolescents from rural areas had superior muscle strength and endurance relative to their counterparts in urban areas. Shen and Huang [28] demonstrated that larger living spaces and living in rural areas were related to higher levels of physical fitness and activity among elementary school-age youth. Chen et al. [29] discovered that adolescents who attended rural schools had higher muscular endurance and flexibility than those who attended urban schools. Their results showed that urban schools provide more playing spaces (i.e., 0.58 square feet per urban student vs. 0.20 square feet per rural student) and exercise equipment than in the rural schools. However, rural schools may have more total spaces (1.22 square feet per urban student vs. 5.09 square feet per rural student) than in the urban schools. Numerous studies have reported that greater space per student indicates a higher likelihood of a student being physically active [28,30,31]. Conflicting results have been reported with respect to the importance of residential area as a determinant of physical fitness and physical activity in adolescents [4,30]. Lower levels of physical activity in urban youth may be due to unsafe neighborhoods, limited access to parks and gyms, and poor facility provision [15,32].

Previous studies on the physical fitness of adolescents have used considerably smaller samples than that used this study. We can conclude that rural students in Taiwanese junior high schools exhibited superior physical fitness relative to students attending urban schools, regardless of gender, and that numerous factors were found to contribute to differences in physical activity. Gender differences were discovered to be significant for all schools, which is consistent with other results [33,34], and indicates that girls from both rural and urban areas must be targeted for priority intervention programs. Schools could be crucial to increasing girls’ physical activity levels if they provided adequate equipment and spaces for activity, and promoted equal gender participation.

Chen et al. [29] reported that urban girls had higher flexibility than did rural girls, which is related to higher muscular endurance; however, no difference was found in boys. The different types of activities boys and girls are encouraged to participate in might help explain why certain factors contributed to their physical fitness. Girls were discovered to be less likely to adopt an active lifestyle unless there was a motivating factor (such as weight loss) or a compelling factor (such as a doctor’s orders).

In this study, we discovered that boys attending schools with a sports field performed well in the indicators of physical fitness, except for flexibility, for which no significant difference was found. Girls attending schools with a sports field had significantly higher muscular endurance, explosive power, and cardiorespiratory ability, but significantly lower flexibility and similar BMI values. School gymnasium provision also contributed to higher physical fitness in both genders. Girls attending schools with a gymnasium had higher flexibility than did those attending schools without. Convenient before-school, in-school, after-school, or weekend access to student-only facilities including playgrounds, multipurpose rooms, and auditoriums, might be essential for the physical activity levels and fitness of adolescents.

One advantage to this study was that selection biases could be eliminated, because the data were from a national survey that was representative of general Taiwanese adolescents. However, several limitations needed to be considered. First, we could not demonstrate causal associations of school environment and after-school physical activity with health-related physical fitness, because this is only a cross-sectional study. Although cross-sectional design is important to understand the relationships between school environment, after-school physical activity, and health-related physical fitness, prospective studies exploring causal association are needed. Second, this study assessed body composition on the basis of BMI. Despite its extensive use to define obesity status, BMI is not an accurate measure of the proportion of fat and fat-free tissue in the body. Percentage of body fat should be considered, and precise measures of body fat should be obtained in future studies.

## 5. Conclusions

Our findings demonstrated that some factors, including urbanization (school location in rural or urban areas), school size, school facility provision (school with or without sports fields or gymnasiums), and after-school physical activity participation are more important than others in shaping adolescents’ physical fitness in Taiwan; meanwhile, these association patterns differed by gender. Our results can provide valuable information to the Taiwanese government in designing the health promoting schools programs for improving the physical fitness and health conditions of adolescents through modifying the exercise environment and participating in after-school exercise interventions. Although further prospective studies are needed, these factors should be taken into consideration when discussing and planning exercise intervention programs for adolescents. In Taiwan, most adolescents prioritize academic work over exercise participation. Therefore, further studies should focus on how after-school exercise can be made to appear more attractive and worthwhile to encourage school-age youth to participate in exercise.

## Figures and Tables

**Table 1:** Demographics of study participants.

Age (Years)	Total	Boys	Girls
13	226,733	118,091	108,642
14	219,343	113,313	106,030
15	203,366	105,673	97,693
Total	649,442	337,077	312,365

**Table 2:** Health-related physical fitness measurements for participants, grouped according to urbanization and school size.

	BMI	Bent-Leg Sit-Ups	800/1600-m Run	Sit-and-Reach	Standing Long Jump
**Boys**					
Rural (*N* = 21,101)	20.7 ± 3.7	37.4 ± 10.3 *	581 ± 141 *	26.0 ± 9.5 *	185 ± 32 *
Urban (*N* = 277,032)	20.7 ± 3.7	36.6 ± 10.1	594 ± 137	25.8 ± 9.4	182 ± 32
**Girls**					
Rural (*N* = 19,249)	20.05 ± 2.96 *	29.9 ± 8.8 *	305 ± 68 *	29.2 ± 9.7 *	145 ± 26 *
Urban (*N* = 260,758)	19.85 ± 2.91	29.4 ± 8.7	303 ± 63	30.2 ± 9.7	144 ± 25
**Boys**					
Large-size school (*N* = 137,484)	20.7 ± 3.6	36.8 ± 9.9 **	598 ± 136 **^,†^	25.6 ± 9.3 **^,†^	182 ± 32 **^,†^
Medium-size school (*N* = 139,090)	20.7 ± 3.7	36.4 ± 10.2 ^‡^	592 ± 138	26.0 ± 9.4 ^‡^	183 ± 32
Small-size school (*N* = 21,074)	20.7 ± 3.7	36.9 ± 10.4	572 ± 142	26.3 ± 9.5	184 ± 32
**Girls**					
Large-size school (*N* = 130,184)	19.8 ± 2.9 **^,†^	29.7 ± 8.6 **^,†^	304 ± 63 **	30.2 ± 9.7 ^†^	144 ± 25 **^,†^
Medium-size school (*N* = 158,789)	19.9 ± 2.9 ^‡^	29.2 ± 8.8 ^‡^	302 ± 63 ^‡^	30.1 ± 9.7 ^‡^	145 ± 25 ^‡^
Small-size school (*N* = 36,839)	20.0 ± 3.0	29.9 ± 8.9	305 ± 69	29.8 ± 9.7	146 ± 25

Notes: BMI = body mass index; SD = standard deviation; Values are expressed as mean ± SD; * Significant difference between rural and urban schools among same-gender adolescents (*p* < 0.05); ** Significant difference between large- and medium-sized schools among same-gender adolescents (*p* < 0.05); ^†^ Significant difference between large- and small-size schools among same-gender adolescents (*p* < 0.05); ^‡^ Significant difference between medium- and small-size schools among same-gender adolescents (*p* < 0.05).

**Table 3:** Health-related physical fitness measurements for participants, grouped according to their schools’ facilities and whether they exercise after school.

	BMI	Bent-Leg Sit-Ups	800/1600-m Run	Sit-and-Reach	Standing Long Jump
**Boys**					
With sport field (*N* = 268,863)	20.68 ± 3.65 *	36.7 ± 10.1 *	593 ± 136 *	25.8 ± 9.4	183 ± 32 *
Without sport field (*N* = 28,785)	20.77 ± 3.66	36.1 ± 10.0	595 ± 147	25.8 ± 9.4	182 ± 32
**Girls**					
With sport field (*N* = 252,201)	19.9 ± 2.9	29.5 ± 8.7 *	303 ± 64 *	30.1 ± 9.7 *	144.3 ± 25.0 *
Without sport field (*N* = 27,399)	19.9 ± 2.9	29.0 ± 8.8	304 ± 65	30.4 ± 9.7	143.8 ± 24.8
**Boys**					
With gymnasium (*N* = 201,726)	20.67 ± 3.63 **	36.7 ± 10.1 **	592 ± 136 **	25.9 ± 9.4	183 ± 32 **
Without gymnasium (*N* = 90,565)	20.73 ± 3.68	36.5 ± 10.0	597 ± 140	25.8 ± 9.3	182 ± 32
**Girls**					
With gymnasium (*N* = 192,148)	19.9 ± 2.9	29.5 ± 8.8 **	303 ± 64 **	30.2 ± 9.7 **	145 ± 25 **
Without gymnasium (*N* = 82,432)	19.9 ± 2.9	29.3 ± 8.5	304 ± 64	30.0 ± 9.6	143 ± 25
**Boys**					
With physical activity after school (*N* = 78,922)	21.1 ± 4.3 ***	36.6 ± 10.1	592 ± 137 ***	26.0 ± 9.4 ***	182 ± 32 ***
Without physical activity after school (*N* = 218,726)	21.2 ± 4.4	36.6 ± 10.1	594 ± 137	25.8 ± 9.4	183 ± 32
**Girls**					
With physical activity after school (*N* = 72,186)	19.8 ± 2.9 ***	29.6 ± 8.7 ***	304 ± 66 ***	30.3 ± 9.8 ***	144 ± 26
Without physical activity after school (*N* = 207,414)	19.9 ± 2.9	29.4 ± 8.7	302 ± 63	30.1 ± 9.7	144 ± 25

* Significant difference between schools with and without sports field among same-gender adolescents (*p* < 0.05); ** Significant difference between schools with and without gymnasium among same-gender adolescents (*p* < 0.05); *** Significant difference between students with and without physical activity after school among same-gender adolescents (*p* < 0.05).

## Abbreviations

The following abbreviations are used in this manuscript:

ANOVA	Analysis of variance
BMI	Body mass index
NCPFS	National Council on Physical Fitness and Sports
NPFS	National Physical Fitness Survey
SD	Standard deviation
